# How Feedback Biases Give Ineffective Medical Treatments a Good Reputation

**DOI:** 10.2196/jmir.3214

**Published:** 2014-08-21

**Authors:** Mícheál de Barra, Kimmo Eriksson, Pontus Strimling

**Affiliations:** ^1^Centre for the Study of Cultural EvolutionStockholmSweden

**Keywords:** bias, social media, behavioral sciences, reputation systems, cultural evolution

## Abstract

**Background:**

Medical treatments with no direct effect (like homeopathy) or that cause harm (like bloodletting) are common across cultures and throughout history. How do such treatments spread and persist? Most medical treatments result in a range of outcomes: some people improve while others deteriorate. If the people who improve are more inclined to tell others about their experiences than the people who deteriorate, ineffective or even harmful treatments can maintain a good reputation.

**Objective:**

The intent of this study was to test the hypothesis that positive outcomes are overrepresented in online medical product reviews, to examine if this reputational distortion is large enough to bias people’s decisions, and to explore the implications of this bias for the cultural evolution of medical treatments.

**Methods:**

We compared outcomes of weight loss treatments and fertility treatments in clinical trials to outcomes reported in 1901 reviews on Amazon. Then, in a series of experiments, we evaluated people’s choice of weight loss diet after reading different reviews. Finally, a mathematical model was used to examine if this bias could result in less effective treatments having a better reputation than more effective treatments.

**Results:**

Data are consistent with the hypothesis that people with better outcomes are more inclined to write reviews. After 6 months on the diet, 93% (64/69) of online reviewers reported a weight loss of 10 kg or more while just 27% (19/71) of clinical trial participants experienced this level of weight change. A similar positive distortion was found in fertility treatment reviews. In a series of experiments, we show that people are more inclined to begin a diet with many positive reviews, than a diet with reviews that are representative of the diet’s true effect. A mathematical model of medical cultural evolution shows that the size of the positive distortion critically depends on the shape of the outcome distribution.

**Conclusions:**

Online reviews overestimate the benefits of medical treatments, probably because people with negative outcomes are less inclined to tell others about their experiences. This bias can enable ineffective medical treatments to maintain a good reputation.

## Introduction

Across cultures and throughout human history, people have sought to alleviate suffering, shorten disease, and alter biological processes using medical treatments. An interesting feature of many medical treatments is that they are not directly beneficial; some even cause significant harm. This is true of Western folk beliefs, alternative medicines [1,2], traditional medicines [3,4], and historical “establishment” medicine like bloodletting [5]. It is also likely to be true of some contemporary medical treatments [6-8]. Treatments may be harmful either to the patient directly or cause harm because they replace other effective treatments, or result in broader environmental harms, as in the case of drugs derived from endangered species [2-4].

Medical treatments are very much cultural traits: rather than being invented anew by each individual, they spread from person to person through cultural processes. The prevalence of poor medical treatments is an anomalous outcome of cultural evolution because culturally acquired information in other domains of life is generally reliable and beneficial. Indeed, the extraordinary ecological success of the human species is, in part, due to our reliance on adaptive cultural information [9]. It is clearly true that humans routinely use cultural information to solve complex problems that, like medicine, entail delayed and/or stochastic feedback. The adaptive value of cultural information is thought to result from a number of mechanisms, such as learning heuristics whereby people selectively imitate more successful people, filtering whereby people evaluate the quality of socially acquired traits through experimentation, and natural selection whereby people with more beneficial cultural traits have more children who then learn these traits [10-12].

Some traditional medicines did have a direct benefit for the patient. Effective variolation, for example, was surprisingly common. For example, Yorba healers in West Africa carried smallpox scabs that could be used to induce a non-lethal infection and resultant immunity [13]. A number of vaccination techniques were being employed in 17th century India and China, and Edward Jenner’s vaccination was long a part of English folk medicine [14]. Some globally important pharmaceutical products have their origins in traditional medicine; Artemisinin, a key anti-malaria drug, was part of ancient Chinese medicine [15]. Moreover, medicine—be it allopathic, traditional, or ancient—is not just about altering the course of disease. Medical experts will often have seen many people with similar diseases and thus they can help patients to understand what their illness is (diagnosis) and how it will play out over time (prognosis). For an anxious patient and his or her family, these are important services and they were probably carried out with some sophistication throughout history and across cultures. Moreover, by identifying and validating illness, medical experts may help the ill to garner social support and thus enable crucial rest and recuperation.

It is also clearly true that patients have undergone surgeries, ingested substances, and been subjected to a litany of other treatments with the explicit expectation that they would be helped. These expectations were not justified: the disease course was unaffected and/or the patient was directly harmed by the treatment. Ineffective treatments were common and remain common, and they warrant study [5]. Why then do harmful and non-beneficial medical treatments spread and persist?

We propose the following explanation. Irrespective of effectiveness, medical treatments typically result in a distribution of outcomes with some people improving, some deteriorating, and others experiencing little change. Suppose that the people who have more positive outcomes are more inclined to tell other people about their experience of the treatment than people who have poorer outcomes. This may occur because people recall their successes better than their failures, because people believe others’ success stories, or because people are embarrassed to have adopted an ineffective treatment. Whatever the cause, such a bias would systematically distort the information available to other naive individuals who are seeking an effective treatment—the reputation of a treatment will exceed its real effect.

This hypothesis is assessed using a variety of methods. First, we compared clinical data on weight loss diets with weight loss reported in reviews of books on these diets. Reviews were taken from Amazon, a popular online marketplace where consumers can post reviews of products. We also made a similar comparison for unproven fertility treatments based on herbs and vitamins. In both cases, we predicted that people with positive outcomes are more inclined to post reviews. In a series of experimental studies, we then tested whether the bias of such reviews is sufficient to influence preferences for treatments. We predicted a preference for weight loss diets accompanied by typical reviews (as sampled from Amazon) over diets accompanied by undistorted reviews (ie, reviews that are representative of the diet’s true effect obtained by purposefully sampling and/or editing of the review). Finally, we used a mathematical model to explore some implications of such reputational distortion.

## Methods

### Study 1: Weight-Loss Diets

In order to make the Amazon and clinical data directly comparable, we made several assumptions and simplifications. Readers interested in conducting alternative analyses or comparisons can access the raw data and analysis syntax from the figshare data repository [16].

The Atkins Diet has been tested in several clinical trials and is the most commonly reviewed diet book on the Amazon online bookstore. We downloaded the 1359 reviews written on or before November 18, 2012. We extracted the duration of the diet and the total weight change from each diet review where this information was provided. If weight change at two time-points was mentioned (eg, 1 kg loss after 1 week and a 3 kg after 1 month), only the longer duration and associated weight change was recorded. If the review described the experiences of more than one person, only information about the author was recorded. If the review only discussed the weight change of a person besides the author, then that person’s weight change was recorded. In total, 587 reviews included both a weight change and a time period over which this change occurred. The median diet duration was 42 days*.* To calculate an average weight loss at 1, 2, 3, 4, 5, 6, 9, and 12 months, we averaged the reports nearest each of these points in time. We excluded reviews of diets that lasted less than 2 weeks or more than 15 months.

**Figure 1:**
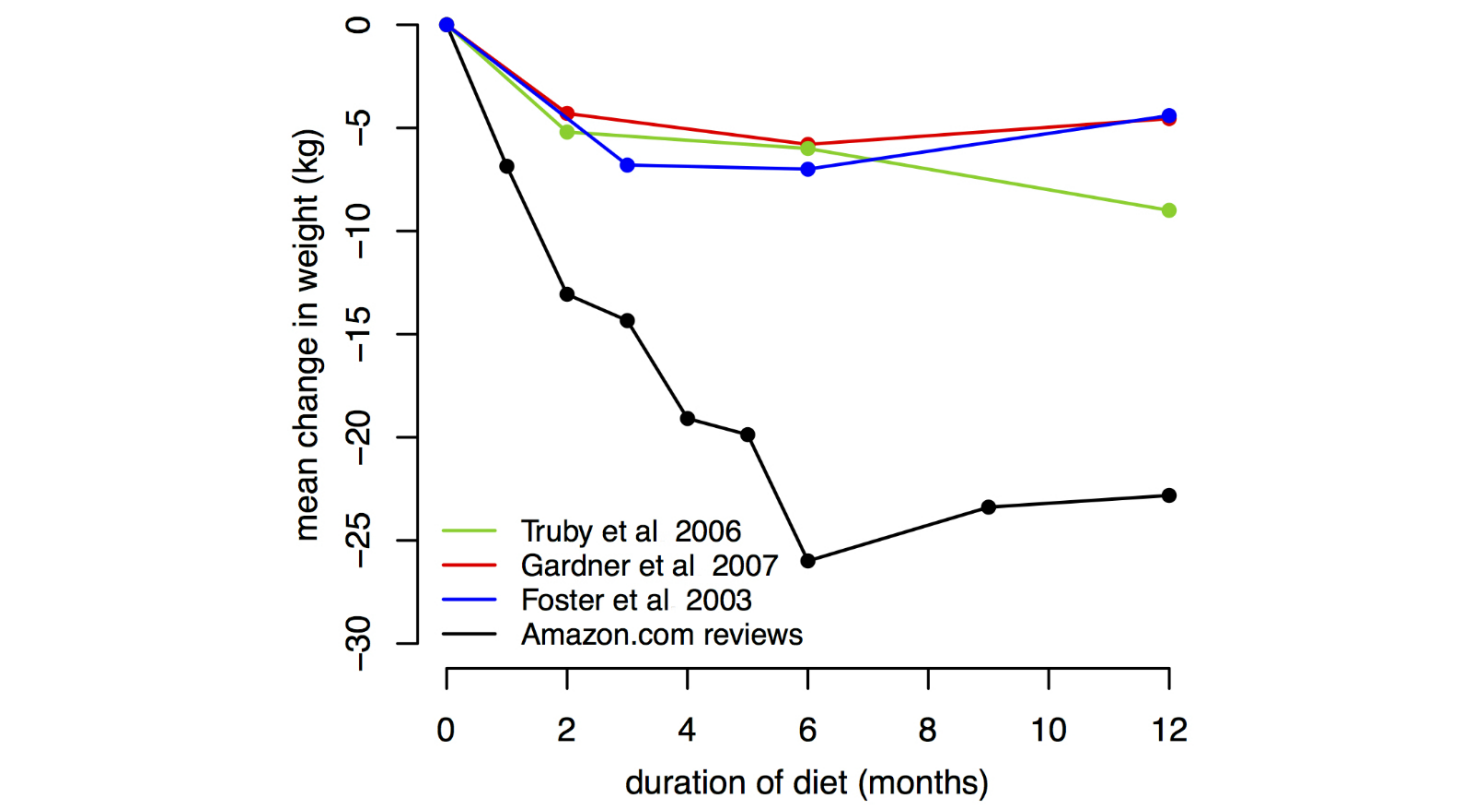
Average weight loss on Atkins diet reported in 3 clinical trials and Amazon reviews. Amazon data points were calculated by averaging reviews nearest the time points 1, 2, 3, 4, 5, 6, 9, 12 months. The numbers of reviews averaged to create the Amazon data points were 129, 60, 60, 23, 22, 19, 26, and 29 respectively.

The “true” effects of the Atkins diet were assessed using three clinical trials [17-19] in which participants received the Atkins diet book. In two of these trials [18,19], the intervention also entailed meeting a dietitian to discuss the diet and the participant’s progress. Basic information about average weight loss in the Atkins diet arm could be extracted from the published manuscript, but to assess the distribution of outcomes, individual level data were needed. Only Gardner et al [18] were willing and able to share their raw data. The Gardner trial examined weight change among 311 premenopausal overweight and obese women, 77 of which were randomly allocated to the Atkins diet. Participants received the Atkins book and met in groups of six once per week for 8 weeks to discuss the diet and book with a dietitian. Although Amazon reviewers are not all premenopausal women, Figure 1 shows that the average effect of the Atkins diet is broadly similar in several different populations. Moreover, given that the intervention involved reading the books *and* meeting with a dietitian, the clinical trial weight loss levels are likely to exceed that found in the general population. We compared the clinical weight change at 2, 6, and 12 months with Atkins reviews written between 1.5 and 2.5 months, 5 and 7 months, and 9 and 15 months respectively.

### Study 2: Fertility Treatments

On May 7, 2013, reviews of FertilAid (n=206), Fertilitea (n=198), and FertilityBlend (n=80) were downloaded from Amazon.com and reviews of Pregnancycare (n=68) were downloaded from Amazon.co.uk (total N=552). These are the most commonly reviewed herbal/vitamin pregnancy pills on Amazon.com and Amazon.co.uk. The following information was extracted from each review, if available: pregnancy status, length of time trying to conceive (TTC) while using the treatment and the length of time TTC before beginning the treatment, presence/absence of a previous pregnancy, the woman’s age, the man’s age, the presence/absence of polycystic ovary syndrome (PCOS), and presence/absence of past pregnancy. Reviews were excluded if the author explicitly stated that pregnancy was not the desired outcome of the treatment.

There is no strong evidence that these treatments enhance fertility in the general population. One pilot study found Pregnancycare was associated with higher pregnancy rates in subfertile/infertile women undergoing ovulatory induction [20] but none of the Pregnancycare reviewers on Amazon reported using Clomid or other ovulatory induction treatments. Another low-power study reports higher pregnancy rates among 53 FertiliBlend users who had previously tried to conceive for 6-36 months [21] but, in the absence of follow-up studies with greater power, it is difficult to ascertain if this difference between treatment groups was clinically meaningful. The National Institute for Health and Care Excellence (NICE) does not recommend any of the aforementioned treatments and notes that “the effectiveness of complementary therapies for fertility problems has not been properly evaluated” [22]. Given the paucity of rigorous data, we assume that these treatments have little effect on fertility.

The pregnancy rates reported on Amazon were compared to pregnancy rates in a prospective study of conception risk in 346 German women [23]. Specifically, pregnancy rates were extracted from data used to generate the Kaplan-Meier survival curves in Figure 1 of that study. The Kaplan-Meier curve corrects for biases due to participant dropout and is considered a best estimate of true pregnancy rate. If women are more likely to write a review after a positive outcome (that is, pregnancy), then conception rates reported in Amazon should be higher than conception rates in the prospective trial. Several important differences between the prospective study and the Amazon data should be noted. First, while the prospective study reports duration TTC in number of cycles, most reviewers report time TTC in days, weeks, or months. Menstrual cycle lengths are quite variable [24] but to enable a direct comparison we assumed one cycle is equivalent to 28 days. Second, women in the prospective study were shown how to use temperature/cervical-mucus monitoring to ensure intercourse occurred on the most fertile days of the cycle. Third, cycles in which intercourse did not occur during fertile days (3%) were excluded from the analysis. Fourth, in the prospective trial, data collection commenced on the month that women switched from oral contraception to “fertility-focused intercourse”. In contrast, of the 153 Amazon reviewers who reported a pre-treatment period trying to conceive, the median period trying to conceive was 1 year. Just 8% of 340 women in the prospective study had not conceived within 12 cycles of fertility-focused intercourse [23]. This indicates that subfertility and infertility is more prevalent among the Amazon reviewers than in prospective study participants. A total of 38 of 558 reviewers (6.9%) reported PCOS, while 83 (14.9%) reported other fertility-related problems (eg, irregular cycles); couples with fertility problems were excluded from the prospective study. Because the prospective study entailed fertility education, exclusion of couples with fertility problems, and the exclusion of cycles where fertile-period intercourse did not occur, the reported conception rate is likely to be higher than what is found in the general population. The comparison between this prospective study and the Amazon reviews is therefore a conservative test of our hypothesis. We are aware of one factor that may bias the results in the other direction: only pregnancies confirmed by a clinician were recorded in the prospective study while any reported pregnancy was included in the Amazon reviews. However, modern digital home pregnancy tests are generally considered reliable.

### Study 3: How Distorted Reputation Influences Treatment Choices

In a series of online experiments, participants recruited from Mechanical Turk, Amazon’s online crowdsourcing marketplace, were presented with two diets and a series of reviews and were then asked to choose between the diets. All participants resided in America, 61% were male and the mean age was 33 years (SD 11). The diet books were *Dr. Atkins Diet New Revolution* and *The 17-Day Diet*. All reviews were extracted from Amazon. Two sets of books/reviews were shown on different pages and the order of presentation was randomized. In one condition, the Atkins reviews were “undistorted” by (1) drawing the reviews from a population of reviews with 200 words or less and an average of 3.5 stars (SD 0.99), corresponding to the average and standard deviation satisfaction rating given to diets in a longitudinal study [25], and (2) adjusting the reported weight change to match the average loss at that time point in clinical trials (calculated using Figure 1). *The 17-Day Diet* reviews were selected randomly from reviews that explicitly stated a weight loss and duration and consisted of 200 words or less (mean number of stars 4.4, SD 0.99). In the other condition, *The 17-Day Diet* reviews had the reputational distortion removed using the same procedure (mean 3.5, SD 1.0), and the Atkins reviews were selected randomly from a sample of reviews that stated duration and weight loss (mean 4.4, SD 1.01). Thus, each book was shown alongside three reviews that were either randomly selected Amazon reviews or purposively selected and edited so as to be consistent with clinical findings. After reading the reviews, participants were asked, “Imagine you decide to begin a diet. Which of these two diets would you begin?”

Ideally, each participant would see a different selection of reviews randomly drawn from the appropriate population. However, technical constraints of our experimental software made this impossible and so instead we ran three versions of each experiment using different reviews randomly selected from the same population. We then averaged the results for these three versions. This procedure was intended to reduce the probability that chance properties of any one set of selected reviews would exert too much influence on the final result. The results were broadly similar across all three versions of the experiment. The results for each condition and the characteristics of the selected reviews are available in Multimedia Appendix 1. Experiment 2 followed the exact same procedure except the diets only differed in positivity—both sets of reviews reported a similar average weight loss. In Experiment 3, the diet reviews were similar in positivity (3.4 stars) but reported different average weight loss. In every case, the dependant variable was diet chosen.

The Act concerning the ethical review of research involving humans (2003:460) regulates research with human subjects in Sweden. Studies need approval only if personal data is collected (ie, race or ethnic origin, political opinions, religious or philosophical beliefs, or membership of a trade union, and data on health or sex life) or if there is an attempt to physically or mentally influence the participant. These studies do not meet these criteria. Participants were clearly informed that by submitting their responses to the questionnaire they consented to the responses being used for research.

## Results

### Study 1: Weight-Loss Diets

In the first study, we compared clinical data on weight loss diets with weight loss reported in reviews of books on these diets. Clinical trials indicate that the Atkins diet results in an average weight change of about −7 kg over the first 6 months and a regain of about 2 kg over the subsequent 6 months [17-19]. In Amazon reviews, the average weight change is about −25 kg after 6 months and −20 kg after 12 months. As Figure 1 shows, the average beneficial effect reported in reviews of the Atkins diet exceeds the real effect at all time points.

In Amazon reviews, weight loss is positively correlated with the number of stars (Spearman’s ρ=.43, *P*<.001), the diet duration (ρ=.71, *P*<.001), the word count (ρ=.14, *P*<.001), the number of capitalized letters (ρ=.1, *P*=.01), but not with the number of exclamation marks (ρ=.05, *P*=.2).

**Figure 2:**
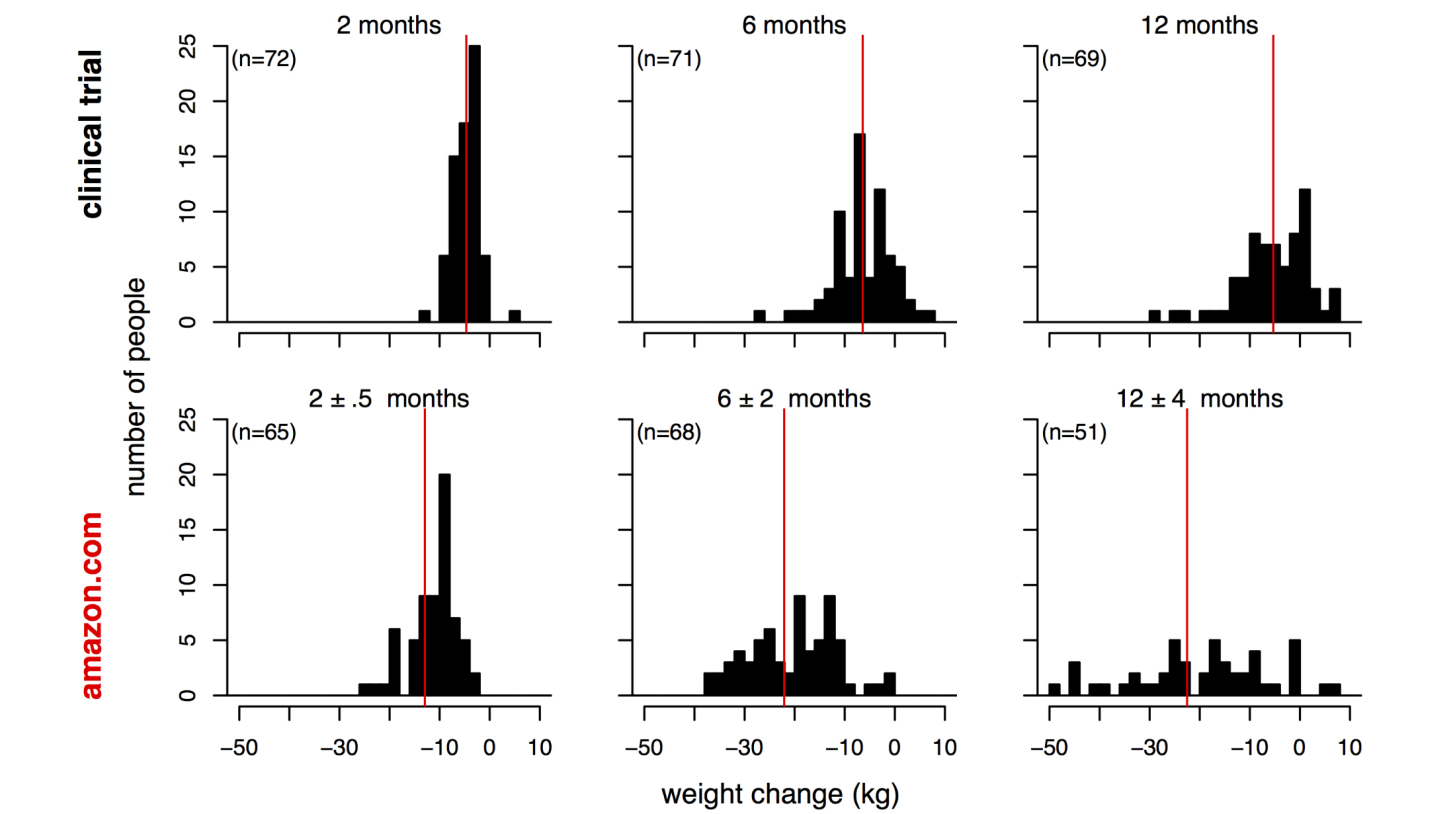
Comparison of weight loss distributions on Amazon reviews (bottom row) and clinical trial (11, top row) at three time points. Horizontal red lines indicate mean weight change. Outliers with weight loss >50 kg are not shown but are included in mean calculation.

Individual level data from a 2007 clinical trial by Gardner et al [18] enabled a detailed comparison of real and reputed effects at three points in time (see Figure 2). The difference between the review data and clinical data was statistically significant at 2 months (*t*_69.8_=5.63, *P*<.001, Cohen’s *d*=0.98), 6 months (*t*_92_=8.72, *P*<.001, *d*=1.48), and 12 months (*t*_60_=5.86, *P*<.001, *d*=1.14). In the clinical trial, participants sometimes lost and then regained weight. The average maximum weight loss for participants in the Gardner trial was 8.33 kg (SE 0.67); this maximum weight loss is also substantially lower than average Amazon weight loss of duration 2 months or greater. These data indicate that while 93% (64/69) of online reviewers reported a weight loss of 10 kg or more, just 27% (19/71) of trial participants experienced a similar weight loss level.

It is possible that the difference between real and reputed weight loss results from fake reviews written by individuals with a vested interest in Atkins sales. Fake reviews are unlikely to be produced continuously over time or at a rate proportional to the number of real reviews. Instead, they should be clustered at strategic times (immediately after an edition of the book is released) or in the period soon after the fake reviews are contracted. Therefore, we examined if the distortion applies over all time periods (suggesting a psychological bias) or if it exists only at certain time periods (suggesting fake reviews drive the distortion). The sample was split into deciles. Each decile contained 50+ individuals, and the deciles spanned from 1996 to 2012. Using the data from Gardner et al, we calculated the predicted weight loss for each participant. Gardner et al provide weight measurement at four time points; weight loss was assumed to be linear between these points. The difference between predicted and actual weight loss was calculated for each participant. A series of 10 one-sample *t* tests showed that in every time period there was a statistically significant distortion (maximum *P* value=.00005). Moreover, the difference between the predicted and real weight loss was of a similar magnitude in each decile (minimum mean difference 6.12, average mean difference 7.56, SD 1.41).

The subset of reviews that include weight change and diet duration information were somewhat more positive than total sample of reviews (mean of 4.43 stars vs 4.06 stars). An alternative explanation for the deviation between the Amazon reviews and the clinical trials results is that people with negative outcomes are less inclined to include specific information about the weight change and duration. In Multimedia Appendix 2, we show that a similar pattern of results is seen when a subset of reviews with a star distribution that matches that of the total sample is analyzed. This alternative hypothesis can therefore be rejected.

### Study 2: Fertility Treatments

**Figure 3:**
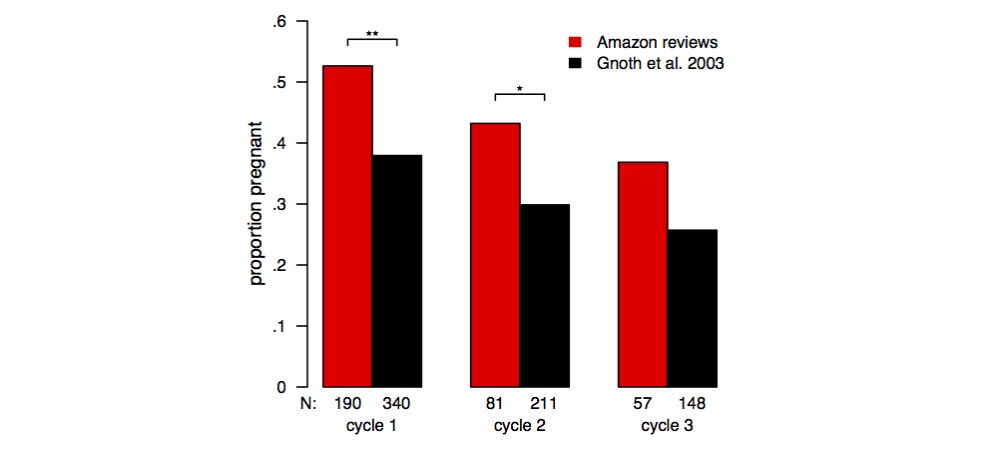
Proportion of non-pregnant women who conceived in each cycle of prospective study and in Amazon reviews of herbal/vitamin fertility treatments. Amazon proportions were calculated by collating reviews in which treatment was used for 28±14 days (cycle 1), 56±14 days (cycle 2), and 84±14 days (cycle 3). 1 star (*) and 2 stars (**) indicate statistically significant differences at P<.05 and P<.01 levels, respectively.

In the second part of our study, we compared fertility data with Amazon reviews of unproven fertility treatments based on herbs and vitamins. In the 552 reviews analyzed, 186 people reported becoming pregnant after taking the treatment, 327 indicated they were not pregnant, and in 39 reviews it was unclear if a pregnancy occurred and/or the reviewer stated that pregnancy was not the desired outcome of the treatment. The duration of the medical treatment was stated in 443 reviews. Excluding the reviews where pregnancy was not reported/desired or the duration of the medical treatment was less than a week, 45.3% (173/382) reported becoming pregnant. Of the women who became pregnant, the median and mean time to pregnancy was 30 and 46 days, respectively. The mean time to pregnancy in the longitudinal study was considerably longer: 3.6 cycles or, if we assume a 28-day cycle, 101 days. Figure 3 illustrates the proportions of Amazon reviewers and study participants who became pregnant in each of the first three menstrual cycles. Chi-square tests indicate that more Amazon reviewers than study participants became pregnant in cycle 1 (100 of 190 vs 129 of 340, *χ* ^*2*^_*1*_=10.04, *P*=.001) and in cycle 2 (35 of 81 vs 63 of 211, *χ* ^*2*^_*1*_=4.70, *P*=.03). In cycle 3, the difference was not statistically significant (21 of 57 vs 38 of 148, *χ* ^*2*^_*1*_=1.97, *P*=.16).

### Study 3: How Distorted Reputation Influences Treatment Choices

Studies 1 and 2 demonstrate that the reputed benefits of medical treatments tend to exceed their actual benefits. The objective of Study 3 was to examine if this reputational distortion is large enough to influence people’s medical decision making.

**Figure 4:**
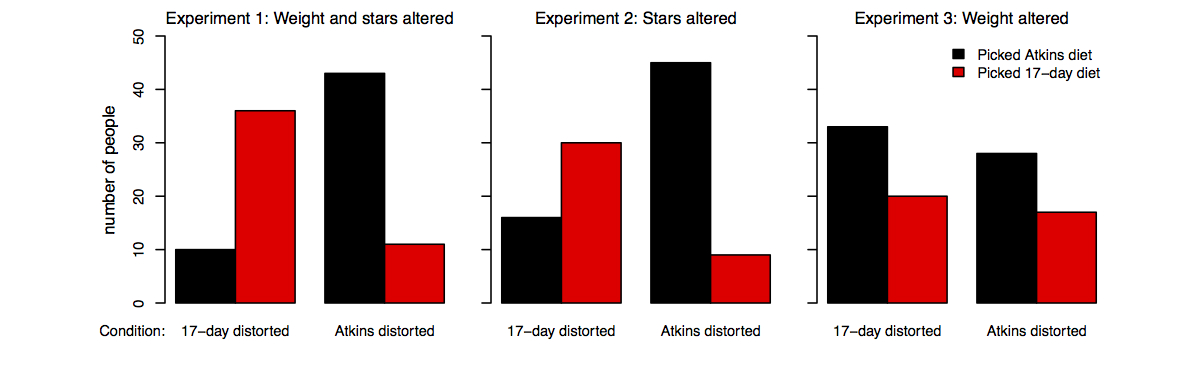
Experiment 1 indicates participants prefer diet book with positive reviews and large weight loss over diet with positivity and weight change more representative of clinical trial results. Experiments 2 and 3 indicate that positivity alone but not weight change alone influence preferences.

Biased reporting can influence cultural evolution if the reputation of the treatment influences subsequent decision. We conducted three experiments with the objective of assessing how positively distorted sets of reviews might influence diet choice. Results indicated that participants were much more likely to pick a diet if its reviews were distorted with respect to both positivity (stars awarded to diet) and weight change (Experiment 1: *χ* ^*2*^_*1*_=33.42, n=100, *P*<.001) or distorted with respect to positivity alone (Experiment 2: *χ* ^*2*^_*1*_=24.61, n=100, *P*<.001). However, reviews that included distorted weight loss alone had no effect on preferences (Experiment 3: *χ* ^*2*^_*1*_=0.02, n=99, *P*=.89). These results are summarized in Figure 4.

### Mathematical Model

**Figure 5:**
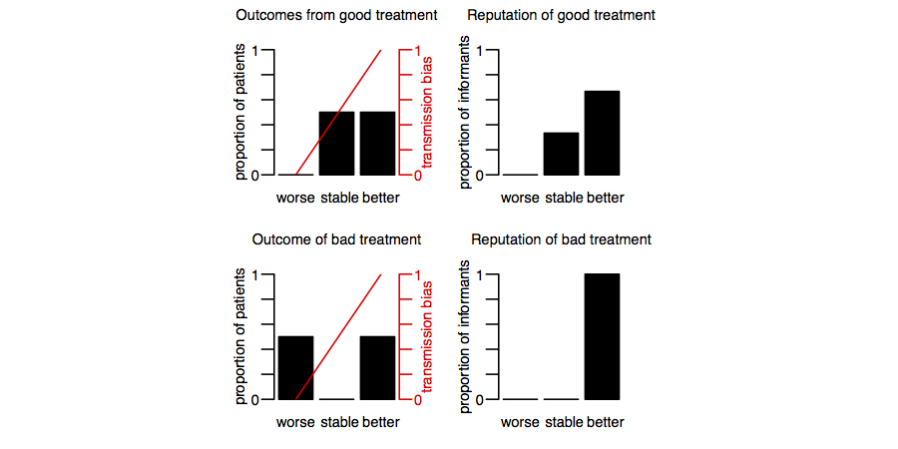
Hypothetical example illustrating the effect explored in the mathematical model. With a reporting bias that makes bad outcomes unobservable, the poorer treatment obtains a better reputation (bottom row: all improve) than the good treatment (top row: 3/4 improve, 1/4 remain stable).

Can this mechanism account for the prevalence of harmful medical treatments across cultures? If the same kind of reporting bias affects all medical treatments, one might think that better treatments will still have a better reputation. However, this is not necessarily the case. Here, we show that the degree to which a treatment’s reputation is distorted by reporting bias will critically depend on the shape of the outcome distribution. In some circumstances, the result will be a superior reputation for an inferior treatment. The basic idea of the model is illustrated in Figure 5.

In order to isolate the effect of the reporting bias, we will make several strong assumptions about how well informed people are. First, we will assume that people have access to an infinite population of informants. These informants are honest, but they are more likely to share information if their outcome is better. Learners then choose the treatment with the best average reputation. This simple model shows that reporting bias can cause the spread of suboptimal treatments in a population.

**Figure 6:**
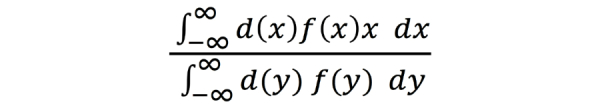
Average observed outcome.

The specific assumptions of the model are as follows: for a focal treatment, let *d*(*x*) denote the density function that describes the distribution of outcomes (measured on some scale of goodness). To implement a reporting bias such that a better outcome is always more likely to be reported than a worse outcome, we assume an individual who obtains outcome *x* will report this outcome with probability *f*(*x*), where *f* is a strictly monotone increasing function of *x.* A learner has access to the reports of an infinite number of people who have tried the treatment in question. The learner then observes a distribution of reported outcomes with density function *d*(*x*)*f*(*x*) divided by a constant factor ∫^*∞*^_−∞_ *d(y) f(y) dy* to maintain unit total probability. Thus, the average observed outcome is shown in Figure 6.

To formalize comparison of treatments, define one treatment as *strictly better* than another treatment if the probability that it gives an outcome better than *x* is always at least as high, and for some *x* higher, than the probability that the other treatment gives an outcome better than *x*. It then holds that for any given treatment, one can always find another outcome distribution, corresponding to a hypothetical treatment, such that the former treatment is strictly better than the latter treatment but nonetheless the learner will choose the latter treatment because it will have a better average observed outcome.

We model goodness of outcomes as values on the real line. Reporting bias is modeled as a strictly monotonic function *f* satisfying *f (x) →* 0 as *x → −*∞ and *f (x) →* 1 as *x →* ∞. Let *d*_1_(*x*) be the density function of a non-degenerate probability distribution on the real line, and let *D*_1_(*x*) denote its cumulative distribution function.

### Theorem 1

**Figure 7:**
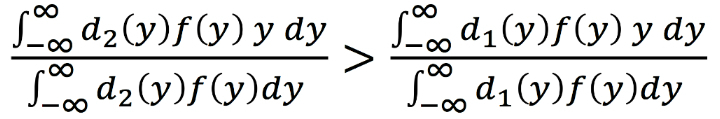
Equation shows treatment two appears more effective.

For every distribution *d*_1_(*x*) with cumulative distribution function *D*_1_(*x*), there exists a distribution *d*_2_(*x*) with cumulative distribution function *D*_2_(*x*) that is strictly worse (ie, *D*_2_(*x*) ≥ *D*_1_(*x*) for all *x* and *D*_2_(*x*) > *D*_1_(*x*) for some *x*) but is perceived as better using some perception bias function *f.* That is the average observed outcome of the strictly worse distribution *d*_2_(*x*) is better than the average observed outcome of *d*_1_(*x*) (Figure 7).

What the theorem says is that there exists a distribution *d*_2_(*x*) of outcomes that is strictly worse than *d*_1_(*x*), but that will nonetheless (under the reporting bias *f*) have higher perceived value (see Multimedia Appendix 3).

## Discussion

### Principal Findings

We found that the reputed benefit of weight loss diets and fertility treatments is larger than the real benefit, apparently because people with typical or poorer outcomes are less inclined to tell others about their experiences. Thus, the real-world reputation of medical treatments seems to be subject to a reporting bias akin to the publication bias toward positive results that is seen in scientific research [26]. Moreover, we found the resultant reputation distortion to be large enough to influence people’s decisions about which diet to begin.

An alternative explanation for the unduly positive reputation of the Atkins diet in our data is that reviewers make mistakes or lie. However, it seems unlikely that measurement error could account for the three- to four-fold difference in weight loss we observed, or that reviewers exaggerate to such a large degree in an online review. Similarly, error alone seems unlikely to account for the significant differences in conception rates, and reviewers had little motivation to lie about pregnancy status. It is also unlikely that fake reviews (written by people wishing to inflate or deflate the reputation of the product) account for our results. The deviation between the reputed benefits and the real effects of the treatments is similar across all eight Atkins diet durations (Figure 1), similar across 15 years of diet book reviews, and is similar over all three menstrual cycles. This consistent pattern of deviation seems more likely to stem from characteristics of human psychology than from deliberate fake review creation.

Although our analysis focused on specific weight change, the experimental data indicates that the general positivity of the review has a stronger influence than the reported weight loss. However, it is not crucial to our main hypothesis whether people are mainly influenced by the emotional or quantitative aspects of others’ experiences because these are closely correlated, both in our data and in other studies of diet satisfaction and weight loss [27-29]. Our sample was perhaps less interested in losing weight than the population of people who are beginning diets. It is possible that prospective dieters would be more sensitive to specific weight information.

### Conditions Where Reputation is Distorted

In summary, we found support for our hypothesis that ineffective and even harmful treatments may spread in a population when (1) treatments depend on word-of-mouth reputation, (2) treated individuals with poor outcomes can remain “invisible”’ if they so wish, and (3) there is a broad range of outcomes. Moreover, the mathematical model shows that the distortion of reputations does not act equally across all treatments: a treatment that succeeds in pulling individuals from bad to intermediate outcomes may, paradoxically, seem worse than a treatment that fails to help individuals with bad outcomes. The bias may therefore account for the historical proliferation of ineffective medical treatments [5].

A slightly different, but conceptually similar, distortion may occur when doctors forget about patients who die under their care. Treatments like bloodletting are especially dangerous to individuals in poor health [30,31]. Given that such individuals were quite likely to remain sick or disabled for the remainder of their lives, a treatment like bloodletting may counter-intuitively appear effective because the past patients who have been bled appear healthier than the past patients who were never bled. What has really happened is that the doctor has “culled” the individuals most likely to remain ill or infirm. Patients killed by harmful treatments may be relatively easy to omit from considerations of treatment effectiveness simply because they have been removed from the community. Although the cause of distortion is different (patients with bad outcomes die and are forgotten versus patients with bad outcomes are inclined to remain silent), our mathematical model describes both cases.

It is not necessarily the case that treatments directly compete in the way our model assumes. Rather than comparing a number of treatments and selecting the one with the best reputation, people may simply adopt the first treatment that meets some criteria (eg, “two consecutive people rate it highly”). The reputational distortion we document means that such criteria will be met more frequently and thus it might cause people to adopt more treatments, including more ineffective ones.

More directly, this feedback bias may be one reason that people have unrealistically high expectations of weight loss diets and other medical treatments. For example, in a study where people were asked to estimate their “dream weight”, “happy weight”, “acceptable weight”, and “disappointed weight”, before they began a 48-week diet, 47% of participants did not even reach their “disappointed” weight [32]. Interestingly, participants’ average “acceptable” weight change was very similar to the average weight change we found reported in Amazon reviews: a 25 kg loss.

This positive distortion in reputation has some important implications for the clinician. Patients are increasingly taking an active role in determining which treatments to adopt. It is unlikely that all the information used to make these decisions will come exclusively from medical professionals or rigorous research: people will listen to their friends, their family, and to other patients with similar experiences. Biases that undermine the reliability of this information, like the one documented here, will become increasingly important. Doctors and patients should be aware of them.

### Conclusions

Researchers have pointed out that several processes make it very difficult to identify benefits and harms of medical treatments when data are not systematically collected. In particular, treatments with no direct effect will sometimes appear effective because of the statistical phenomenon known as regression to the mean and the physiological phenomenon known as the placebo effect [33,34]. It has also been suggested that treatments that prolong illness may, perversely, spread better because they are “demonstrated” for a longer period than effective treatments [35]. Here, we have explored an additional mechanism, reporting bias, and its logical consequence: when people with poor outcomes remain silent, the reputed benefit of a treatment will exceed its real effect.

## Multimedia Appendix 1

Details of stimuli and results of experiments in Study 3.

## Multimedia Appendix 2

Analysis of a subset of reviews with star distribution equal to that of total review sample.

## Multimedia Appendix 3

Proof of mathematical theorem.

## Abbreviations

PCOS: polycystic ovary syndrome
TTC: trying to conceive
