# Supplementary material for: How Feedback Biases Give Ineffective Medical Treatments a Good Reputation
Source: J Med Internet Res. 2014 Aug 21;16(8):e193. doi: 10.2196/jmir.3214 (PMC4147705; doi:10.2196/jmir.3214)
Supplement: Supplementary file 1 [file jmir_v16i8e193_app1.pdf]

Experiment 3: Methods

In the table below you can see the characteristics of the reviews shown to participants in each of the six conditions.

**Exp 1: KG & Stars**		Atkins			17-day		
		Star	LB change	Duration	Star	LB change	Duration
Atkins>17	Exp1	4	-27	60	4	-10	60
		5	-11	23	2	-5	23
		5	-4	8	4	-1.5	8
	Exp2	5	-27	60	5	-10	60
		5	-8	17	3	-3	17
		3	-3	6	3	-1	6
	Exp3	5	-32	90	4	-12	90
		5	-2	5	5	-1	5
		3	-3	5	3	-1	5
17>Atkins	Exp1	4	-11	75	5	-30	75
		3	-6	28	5	-13	28
		4	-0.5	2	5	-1	2
	Exp2	3	-3.5	17	4	-8	17
		4	-1	7	5	-3	7
		2	-1	6	5	-2.5	6
	Exp3	3	-6	30	5	-14	30
		4	-3	14	4	-7	14
		3	-2.5	12	3	-6	12
**Exp 2: Stars**		Atkins			17-day		
		Star	LB change	Duration	Star	LB change	Duration
Atkins>17	Exp1	5	-7	14	4	-2	15
		5	-15	34	4	-15	34
		4	1	5	3	1	5
	Exp2	5	-3	14	3	-3	12
		5	-20	210	5	-20	210
		5	-40	90	3	-40	90
	Exp3	5	0	14	2	0	14
		3	0	14	3	0	14
		5	-11	14	3	-11	14
17>Atkins	Exp1	3	-6	10	4	-6	10
		5	-5	6	5	-5	6
		2	-10	7	5	-10	7
	Exp2	3	-20	30	5	-20	30
		5	-19	35	5	-19	34
		5	-32	49	4	-32	49
	Exp3	4	-9	14	5	-9	14
		4	-20	60	5	-21	60
		3	-16	98	5	-15	100
**Exp 3: KG**		Atkins			17-day		
		Star	LB change	Duration	Star	LB change	Duration
Atkins>17	Exp1	4	-1	2	2	0	2
		4	-7	14	2	-2.5	14
		5	-20	45	3	-8	45
	Exp2	3	-4	8	3	-1.5	8
		3	-27	60	5	-10	60

		3	-10	21	5	-4	21
	Exp3	2	-3	7	3	-1	7
		3	-7	14	4	-2.5	14
		4	-30	75	5	-11	75
17>Atkins	Exp1	4	-0.5	2	2	1	2
		4	-2	14	2	-7	14
		5	-8	45	3	-21	45
	Exp2	3	-1	8	3	-4	8
		3	-10	60	5	-27	60
		3	-3.5	21	5	-10	21
	Exp3	2	-1	7	3	-3	7
		3	-2.5	14	4	-7	14
		4	-11	75	5	-30	75

Experiment 3: Results

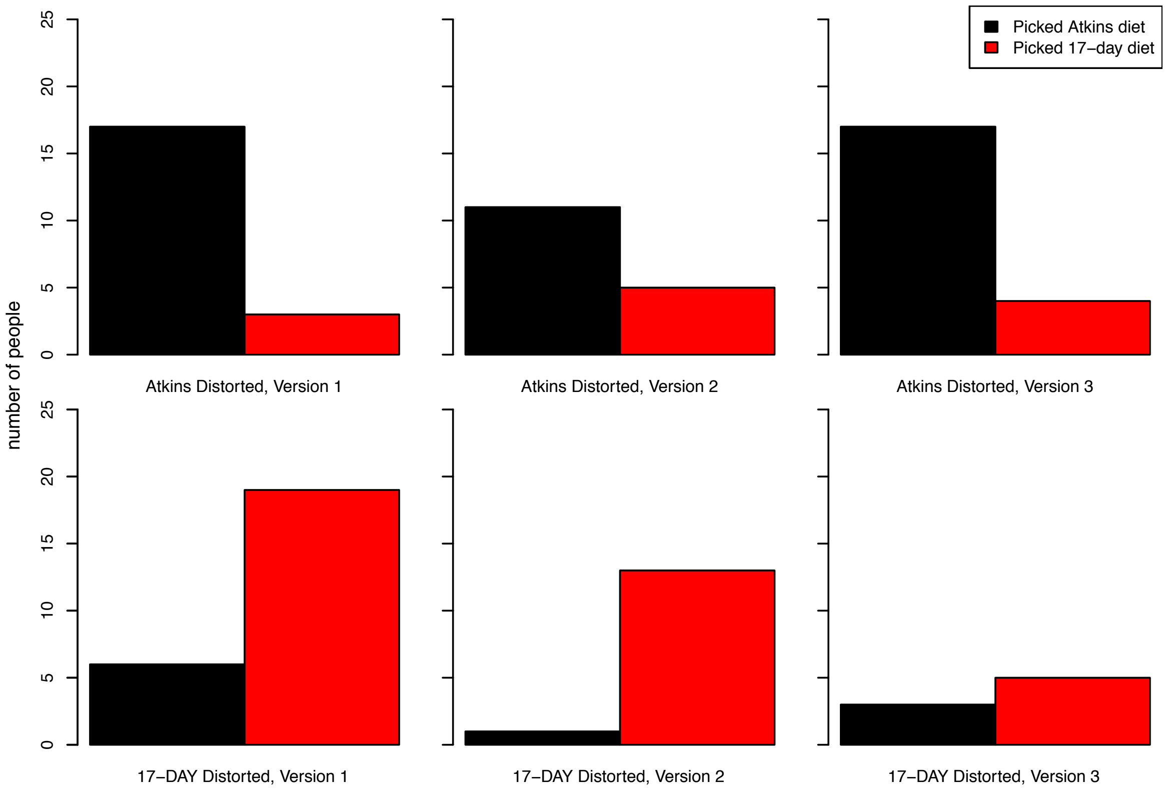

Figure 1: Results of experiment 1, versions 1 to 3.

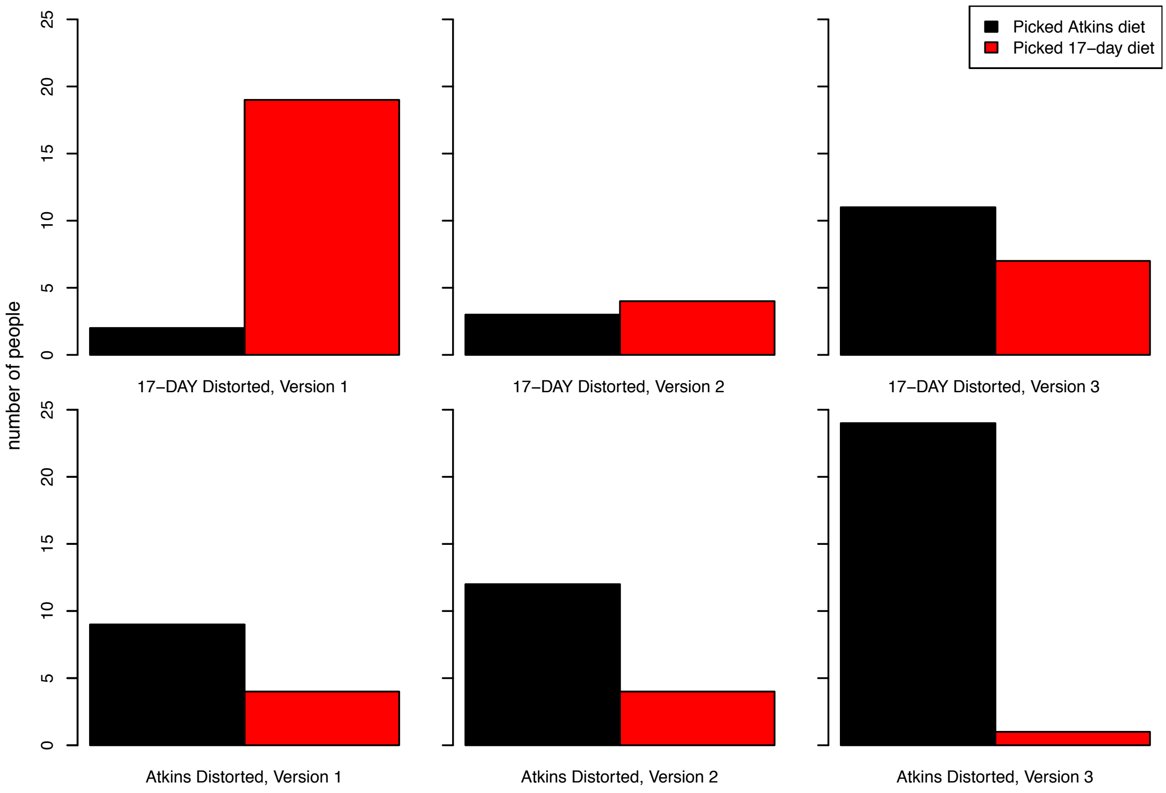

Figure 2: Results of experiment 2, versions 1 to 3.

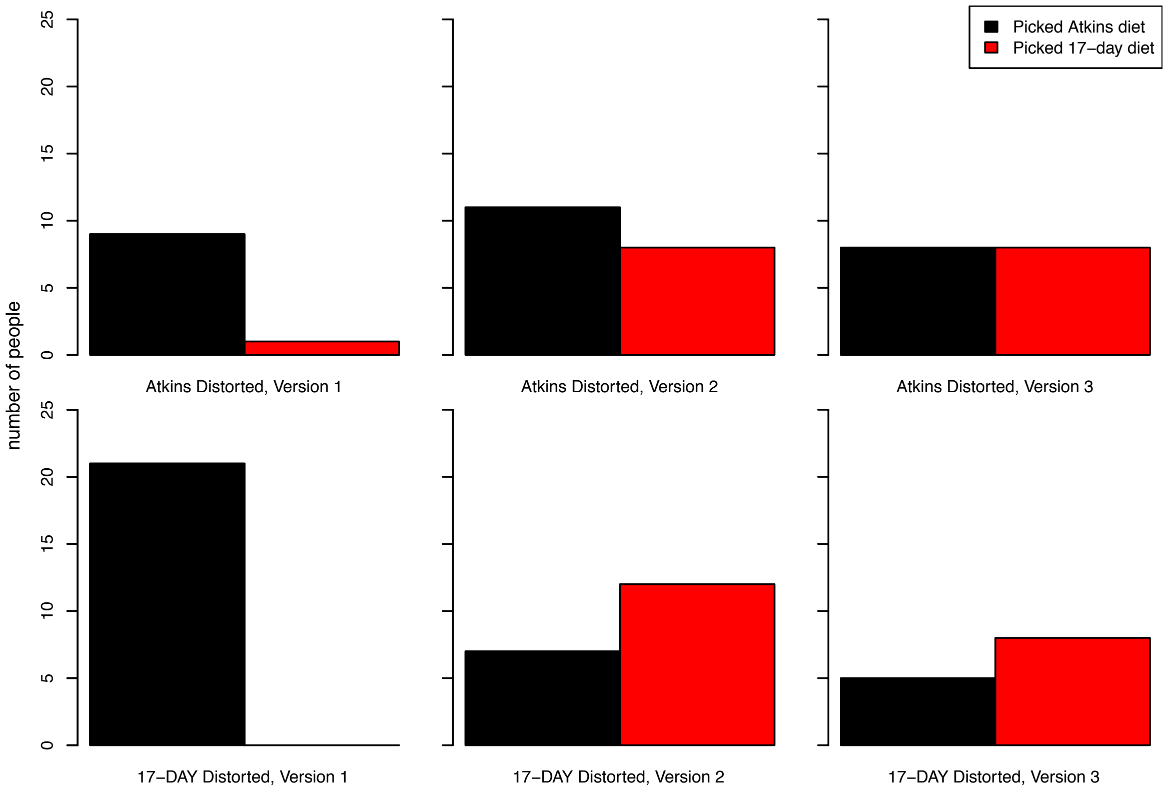

Figure 3: Results of experiment 1, versions 1 to 3.
