# Supplementary material for: How Feedback Biases Give Ineffective Medical Treatments a Good Reputation
Source: J Med Internet Res. 2014 Aug 21;16(8):e193. doi: 10.2196/jmir.3214 (PMC4147705; doi:10.2196/jmir.3214)
Supplement: Supplementary file 2 [file jmir_v16i8e193_app2.pdf]

**Multimedia Online Materials 1:**

The proportion of negative reviews in the analyzed sample is lower than the proportion of negative is the total sample of reviews; see top and middle panel in figure 1 below:


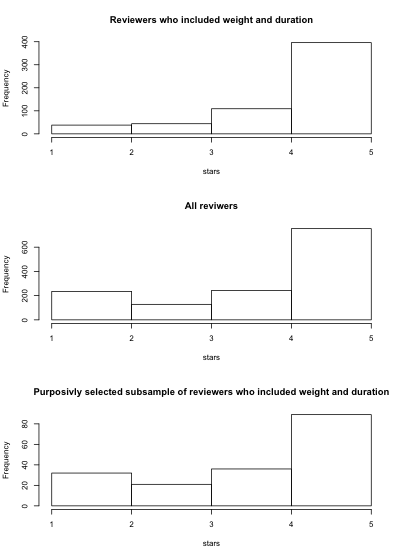


Figure 1: Number of stars awarded to diet books by reviewers who included weight change and duration (top panel) and by all reviewers (middle panel) and by subset of reviews included weight and duration (bottom panel). See text for details of subset.

An alternative explanation for the deviation between the amazon reviews and the clinical trials results is that people with negative outcomes are less inclined to include specific information about the weight change and duration. In other words, by just focusing on the reviews that include both a weight change and a duration, we ignore many negative reviews and this accounts for the distorted reputation. We tested this hypothesis using a new sample of reviews that include weight/duration and with a star distribution that matches the total sample.

For each star category, we calculated a probability such that if reviews in the star category were selected with this probability, the resultant distribution would match that of the total sample (All reviews in fig 1). A subset of reviews were randomly selected at this probability resulting in a new subset of reviews. See the bottom panel in figure 1.

The major analyses presented in Study 1 were repeated using this subset.

Table 1: Characteristics of total sample and purposively selected subsample

|  | All reviews with duration and weight change | Purposively selected subset |
| --- | --- | --- |
| N | 587 | 178 |
| Mean Stars | 4.43 | 3.90 |
| Mean duration (days) | 166 | 167 |
| Mean weight change (kg) | -11.97 | -10.01 |
| Mean word count | 186 | 171 |

The difference between the review data and clinical data extracted from Gardner et al. was statistically significant and in the predicted direction at 2 months (t = 5.41, df = 13.96, *p* < .001, Cohen’s d = 1.84), 6 months (t = 3.34, df = 15.73, *p* = .004, Cohen’s d = 1.13) and 12 months (t = 2.39, df = 15.5, *p* = .03, Cohen’s d = 0.84).

Thus, even when we take a randomly selected subset with a star distribution similar to that of the overall sample, the difference between the Amazon reviews and the clinical trial remains statistically significant.
