# Supplementary material for: How Feedback Biases Give Ineffective Medical Treatments a Good Reputation
Source: J Med Internet Res. 2014 Aug 21;16(8):e193. doi: 10.2196/jmir.3214 (PMC4147705; doi:10.2196/jmir.3214)
Supplement: Supplementary file 3 [file jmir_v16i8e193_app3.pdf]

Multimedia Appendix 3

Theorem 1. For every distribution $d_1(x)$ with cumulative distribution function $D_1(x)$ there exists a distribution $d_2(x)$ with cumulative distribution function $D_2(x)$ that is strictly worse (i.e. $D_2(x) \geq D_1(x)$ for all x and $D_2(x) > D_1(x)$ for some x) but is perceived as better using some perception bias function *f*. That is the average observed outcome of the strictly worse distribution $d_2(x)$ is better than the average observed outcome of $d_1(x)$ i.e. $\frac{\int_{-\infty}^{\infty} d_2(y)f(y)\,y\,dy}{\int_{-\infty}^{\infty} d_2(y)f(y)dy} > \frac{\int_{-\infty}^{\infty} d_1(y)f(y)\,y\,dy}{\int_{-\infty}^{\infty} d_1(y)f(y)dy}$.

What the theorem says is that there exists a distribution $d_2(x)$ of outcomes that is strictly worse than $d_1(x)$, but which will nonetheless (under the reporting bias *f*) have higher perceived value.

Proof: Because $d_1(x)$ is a non-degenerate distribution, we can pick a point g such that $\int_{-\infty}^{g} d_1(x)\,dx > 0$ and $\int_{g}^{\infty} d_1\,(x)dx > 0$. For a large positive number z, define

$$d_2(x) = \begin{cases} d_1(x) & \text{for } x \geq g \\ 0 & \text{for } g > x \geq g - z. \\ d_1(x+z) & \text{for } g - z > x \end{cases}$$

We first show that $D_2(x) \geq D_1(x)$ for all x and $D_2(x) > D_1(x)$ for some x. For $x \geq g$,

$$D_2(x) = \int_{-\infty}^{x} d_2(y)\,dy = \int_{-\infty}^{g-z} d_1(y+z)dy + \int_{g}^{x} d_1(y)dy = \int_{-\infty}^{x} d_1(y)dy = D_1(x)\,.$$

For $g > x$,

$$D_2(x) = \int_{-\infty}^{x} d_2(y)dy = \int_{-\infty}^{\min\{g,x+z\}} d_1(y)dy > \int_{-\infty}^{x} d_1(y)dy = D_1(x).$$

Next we show that for a sufficiently large z the perceived value of d_2 is larger than the perceived value of d_1. The perceived value of d_2 is

$$\frac{\int_{-\infty}^{\infty} d_2(y)f(y)\,y\,dy}{\int_{-\infty}^{\infty} d_2(y)f(y)dy} = \frac{\int_{g}^{\infty} d_1(y)f(y)\,y\,dy + \int_{-\infty}^{g-z} d_1(y+z)f(y)\,y\,dy}{\int_{g}^{\infty} d_1(y)f(y)dy + \int_{-\infty}^{g-z} d_1(y+z)f(y)dy}$$

Because $f(x) \to 0$ when $x \to -\infty$, both $\int_{-\infty}^{g-z} d_1(y+z)f(y)\,y\,dy$ and $\int_{-\infty}^{g-z} d_1(y+z)f(y)dy$ tends to zero as z grows to infinity. For sufficiently large z the perceived value of d_2 will therefore be arbitrarily close to

$$\frac{\int_{g}^{\infty} d_1(x)f(x)\,x\,dx}{\int_{g}^{\infty} d_1(y)f(y)dy}$$

which is the perceived value of the top part of distribution d_1 and therefore always higher than the perceived value of the whole of d_1.

Q.E.D.
